# Combinatorial Design of Slippery, Nitric Oxide-Releasing Surfaces Incorporating Copper Nanoparticles for Blood-Contacting Devices

**DOI:** 10.1021/acsami.6c02385

**Published:** 2026-04-27

**Authors:** Vicente D. Pinon, Mark Garren, Yi Wu, Grace H. Nguyen, Elizabeth J. Brisbois, Hitesh Handa

**Affiliations:** † Department of Pharmaceutical & Biomedical Sciences, 1355 University of Georgia, Athens, Georgia 30602, United States; ‡ School of Chemical, Materials, and Biomedical Engineering, College of Engineering, 1355 University of Georgia, Athens, Georgia 30602, United States

**Keywords:** nitric oxide, liquid infusion, copper nanoparticles, antibacterial

## Abstract

Hospital acquired infections (HAIs) remain a prevailing issue in clinical settings. These challenges are associated with the health and economic burdens of complications such as bloodstream infections, biomedical device fouling, and antimicrobial resistance. Nitric oxide (NO) is an endogenous gasotransmitter that has vasodilatory, antimicrobial, and antiplatelet effects. This has allowed NO to surface as a potential bioactive strategy for integration into medical device technologies. When combined with other surface modifications, such as liquid infusion (LI) and copper nanoparticles­(CuNPs), this approach yields slippery, antifouling surfaces with dual antibacterial and NO-catalysis properties. Herein, we combined *S*-nitroso-*N*-acetylpenicillamine (SNAP), a promising nitric oxide donor that releases NO at physiological levels for 7 days in the presence of heat and light, with catalytic tuning via CuNPs with a LI polymeric surface. In addition, SNAP Cu LI samples exhibited minimal donor leaching and slippery behavior with sliding angles of <20° across a 7-day test period. These propertiesof NO support its antimicrobial and antiplatelet effects in addition to enhanced catalysis induced by metal–donor interactions. This eradicates microorganisms such as methicillin-resistant *Staphylococcus aureus* (MRSA) and *Escherichia coli* with >99.8% and 98.7% reduction in bacterial cell viability, respectively. In addition, LI reduced blood protein adsorption by >80% and prevented platelet activation by >72%. By integrating nitric oxide-releasing silicone rubber with copper nanoparticles and a liquid-infused layer, these surface modifications produce a multifunctional medical device surface with antibacterial, antithrombotic, and antifouling capabilities.

## Graphical abstract

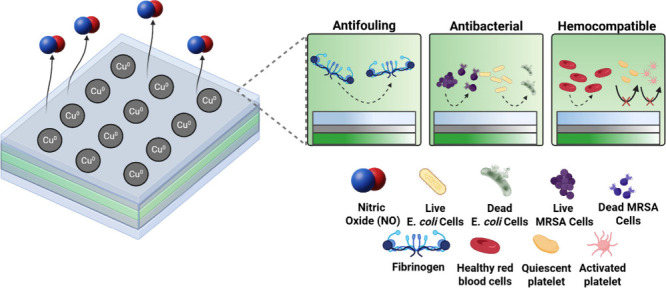

## 1 Introduction

Indwelling and implanted devices are a crucial component of modern healthcare. Broad applications of these devices include urinary catheters, respiratory ventilators, heart valves, and central line venous catheters, which are exposed to biological systems and environments for hours to years at a time.^1^ This exposure may result in prevalent complications within the clinical setting. These complications often stem from disruption of normal tissue and blood interfaces, which can ultimately attract pathogen colonization, protein adsorption, platelet activation, and thrombus formation. These complications impair device functionality, compromise patient safety, and increase the risk of Hospital Acquired Infections (HAIs).^2^ HAI’s affect 1.7 million patients and can have an economic impact of up to $33.8 billion on U.S. hospitals annually.^3^ Current treatments address these complications through the administration of various antibiotics and anticoagulant drugs, such as established heparin or a range of antibiotics, including vancomycin, daptomycin, and amikacin.^4^ However, complications such as antibiotic-resistant bacteria and biofilm formation, often stemming from the treatment’s mechanism of action, along with limitations of anticoagulant therapies, including poor patient response, toxicity, and lack of specificity, have reduced the effectiveness of these treatments.^5^ This highlights the urgent need for continued research into alternative therapeutic strategies. Nitric oxide (NO) is an endogenous gasotransmitter that is recognized to play roles in wound healing, vasodilation, antimicrobial activity, and biological stabilization.^6^ This molecule is biologically synthesized via arginine-dependent pathways through NO synthase enzymes, including endothelial nitric oxide synthase (eNOS), neuronal nitric oxide synthase (nNOS), and inducible nitric oxide synthase (iNOS).^6^ NO is released from endothelial cells at a rate of 0.5–4.0 × 10^–10^ mol min^–1^ cm^–2^ to regulate muscular vasculature and prevent platelet activation.^7^ In recent decades, the application of NO in biomedical research has increased substantially, and new properties have been further researched, making NO a highly renowned possibility for addressing various medical applications.^8–10^

Through the discovery of these applications, NO has been integrated into various medical device applications through the implementation of NO donor molecules such as synthetic and endogenous *S*-nitrosothiols (RSNOs) and *N*-diazeniumdiolates (NONOates) that provide stable NO release into a system at therapeutic concentrations, which further enhances its beneficial physiological roles through the synergistic effect between these surface modifications and the biological system.^11–14^

*S-*nitroso-*N*-acetylpenicillamine (SNAP), a nitric oxide donor, has proven effective for direct integration into biomedical devices owing to its controlled NO release.^12,15,16^ It also shows strong cytocompatibility and exhibits antimicrobial and antithrombotic effects in diverse biological evaluations.^12,16,17^ This molecule releases NO through cleavage of the S-NO bond in the presence of light, heat, and interaction with metal ions. Depending on surface interactions, SNAP has been shown to release NO via polymeric interfaces, and its applications are broad, as NO release can range from hours to days or months.^17,18^ Further combination strategies to enhance the role of these molecules have enabled the development of promising clinical solutions to address complications associated with indwelling medical devices. Recent advances in NO research demonstrate that combination strategies employing NO donor molecules with metal ions, such as copper nanoparticles (CuNPs), enable controlled NO release within the system to maximize the molecule’s benefits while avoiding cell toxicity.^19,20^ The role of CuNPs also enhances antimicrobial properties, thereby further reducing bacterial adhesion to the surface of a medical device.^21,22^ Copper ions possess contact-killing mechanisms via bacterial cell wall permeation and induction of cellular stress through reactive oxygen species (ROS) production.^21^ In addition, this metal exhibits dual functionality as an NO donor and in biological systems via a reaction mechanism that interacts with thiols, thereby reducing Cu^2+^ to Cu^+^. This reduction, in the presence of RSNOs, allows NO to be produced while oxidizing Cu back to its Cu^2+^ state.^23^ This conversion reinitiates this cycle, providing a nitric oxide-generating mode of action that works synergistically with both the material surface that is releasing NO and endogenous RSNOs.^20,24^ The combination of SNAP and copper nanoparticles (CuNPs) offers a promising approach to reducing infection and clotting. SNAP releases nitric oxide (NO), which inhibits bacterial growth and prevents platelet activation, whereas CuNPs enhance this effect by catalyzing NO release. However, these strategies are limited by surface fouling resulting from initial protein adsorption upon material-blood contact. Implementing a low-fouling surface modification is crucial for enhancing the biocompatibility of blood-contacting medical devices.

Development of ultralow fouling surfaces stem from natural approaches such as the *Nepenthes* pitcher plant, when Slippery Liquid Infused Porous Surfaces (SLIPS) were first classified by Wong et al.^25^ Liquid infusion (LI) via silicone oil swelling into the polymeric matrix of these devices yields a surface that is antifouling by creating a slippery interface that prevents blood protein adsorption as well as enhancing bacterial repellence properties.^26^ In the presented work, a 3-dimensional class of liquid-infused surfaces that uses a silicone rubber substrate, specifically polydimethylsiloxane (PDMS), to swell and lubricate the polymer surface was used.^27^ This method optimizes medical device surfaces to prevent surface fouling caused by biological agents and facilitates the integration of slippery surface modifications. This study presents a novel strategy for addressing clinical challenges by combining the nitric oxide donor SNAP with copper nanoparticles (CuNPs) and liquid infusion. This multifunctional approach effectively combats bacterial infections and targets thrombus formation at multiple stages, including protein adsorption and platelet activation during coagulation. The resulting surface is an antifouling, biocompatible, liquid-infused, copper-coated, NO-releasing substrate (SNAP Cu LI) that demonstrates how a combinatorial design can advance the optimization of medical device surfaces.

In the presented work, a multifunctional surface technology was evaluated by integrating active NO release for antimicrobial and hemocompatible effects, catalysis and antibacterial activity using CuNPs, and antifouling behavior through integration of silicone oil. From these surface modifications, enhanced antibacterial properties, reduced blood protein adsorption, and improved hemocompatibility are achieved compared with uncoated silicone rubber substrates. These SNAP Cu LI surfaces were fabricated using a facile multistep fabrication process via dip coating and oil swelling methods allowing the components to combine into the polymeric matrix. The experimental surfaces were thoroughly characterized for their NO donor stability, sliding angle, and NO release kinetics. In addition, *in vitro* biological studies were conducted against clinically relevant bacterial species, while studies against mouse fibroblast cells were conducted to evaluate the cytocompatibility of the material. Lastly, blood interactions such as platelet adhesion and hemolysis were explored. Antifouling properties of SNAP Cu LI were also assessed in fibrinogen adsorption studies. Developing surfaces that effectively combat issues like surface fouling, bacterial infections, and thrombosis—while remaining nontoxic—can profoundly advance the future of medical device innovation.

## 2 Materials and Methods

### 2.1 Materials

Phosphate buffered saline (10 mM, pH 7.4) comprising of potassium chloride (2.68 mM), sodium chloride (139 mM), sodium phosphate monobasic (1.8 mM), and sodium phosphate dibasic (8.2 mM) was obtained from Fisher BioReagents (Hampton, NH) and adjusted to desired pH with a Mettler Toledo SevenCompact pH/Ion Meter (Colombus, OH). Copper nanoparticles (Cu, 99.9%, 40–60 nm) were purchased from Skyspring Nanomaterials (Houston, TX). Dowsil 3140 RTV silicone rubber was obtained through Ellsworth Adhesives (Germantown, WI). Reagents, including tetrahydrofuran (THF), methanol (MeOH), sodium nitrite (NaNO_2_), sulfuric acid (H_2_SO_4_), silicone oil (10 and 50 cSt), hydrochloric acid (HCl), LB broth and agar, copper detection kit, and sodium citrate dihydrate were purchased from Sigma-Aldrich (St. Louis, MO). Calcium and magnesium-free phosphate buffered saline (CMF-PBS, 1×) was purchased from Corning Incorporated (Corning, NY). Bacterial strains *Escherichia coli* (ATCC 25922) and methicillin-resistant *Staphylococcus aureus* (MRSA BAA41), as well as human BJ fibroblasts (ATCC CRL-2522) were obtained from the American Type Culture Collection. Drabkin’s Reagent was bought from Ricca Chemical Company (Arlington, TX).

### 2.2 Fabrication of SNAP Cu LI

#### 2.2.1 SNAP Synthesis

SNAP was synthesized utilizing a previously reported method through the nitrosation of the precursor molecule N-acetyl-penicillamine (NAP).^28^ In brief, a sodium nitrite solution was combined with NAP, methanol, 95% H_2_SO_4_ in DI H_2_O, and concentrated HCl and allowed to stir under N_2_ gas to achieve a nitrosated product. SNAP crystals were collected through vacuum filtration and washed with DI H_2_O to remove unreacted sodium nitrite and other byproducts. The final SNAP product was dried overnight under vacuum desiccation and used for film fabrication. Purity testing was conducted using nuclear magnetic resonance (NMR) spectroscopy, yielding >95% purity via chiral center integration of SNAP and its disulfide byproduct, confirming SNAP’s viability for experimental use.

#### 2.2.2 Fabrication of Experimental Films

Fabrication of samples used for characterization and biological studies commenced by combining a clinically relevant silicone rubber (SR) substrate, specifically Dowsil 3140 Room Temperature Vulcanizing (RTV) silicone rubber, with tetrahydrofuran (THF) at a concentration of 250 mg mL^–1^, which was then cured overnight to yield control SR samples. For SR Cu samples, SR films were dip-coated with one barrier coat of SR, followed by three coats of copper nanoparticles (3 wt %) in a 100 mg mL^–1^ SR solution. SR LI samples were incubated in 10 cSt and 50 cSt silicone oil for 24 h to yield a slippery SR LI material. For the NO-releasing sample types, RTV was combined in THF at a concentration of 250 mg mL^–1^. SNAP was blended at 10 wt % (375 mg SNAP) and cast into a 7 × 7 cm Teflon mold overnight for curing. Subsequently, these bulk films were punched out into 6 mm disks with a biopsy punch for dip-coating and testing. SNAP films without CuNPs or LI surface modifications were dip-coated with 4 coats of SR (100 mg mL^–1^) to ensure a consistent topcoat thickness. SNAP Cu films were dip-coated with one barrier coat of SR, followed by three coats of copper nanoparticles (3 wt %) in a 100 mg mL^–1^ SR solution. Finally, SNAP Cu LI samples were fabricated using the same methods as SNAP Cu films and incubated in 10 and 50 cSt silicone oil for 24 h to yield a slippery, NO-releasing copper-coated polymer platform for subsequent testing.

### 2.3 Material Characterization

#### 2.3.1 SNAP Leaching Analysis

To analyze depletion of the SNAP reservoir loaded in SNAP Cu LI samples, a 24 h cumulative SNAP leaching analysis was performed using UV–vis spectroscopy. Film samples were punched into 8 mm disks and exposed to 1 mL of PBS buffer, pH 7.4, at 37 °C in dark conditions to mimic a physiological environment. SNAP saturation in the PBS solution was analyzed at various time points, including 0, 1, 4, 8, 24 h, by aliquoting 1 mL of incubation solution and quantifying the absorbance at the wavelength of SNAP (340 nm). SNAP leaching was quantified against a standard curve of known concentrations of SNAP in PBS and normalized to the weight of SNAP (mg) with respect to surface area of the samples (cm^2^).

#### 2.3.2 Copper Leaching

To assess potential effects of copper accumulation in the physiological environment and biological studies, a colorimetric copper leaching study was conducted on SNAP Cu LI samples using a copper assay kit (Sigma-Aldrich, St. Louis, MO). For analysis, 6 mm Cu-containing sample disks were incubated in PBS for 24 h at 37 °C in dark conditions. After incubation, 100 μL of the sample solutions was added to a 96-well microplate for analysis. The copper detection assay was prepared using a master reaction mix according to the manufacture’s protocol and added to each well containing incubation solution, followed by incubation. After incubation, samples were read using a BioTek Cytation 5 microplate reader (Shoreline, WA) at 359 nm. Final values were quantified using a standard curve prepared using the manufacturer’s standard range of 0–300 Cu^2+^ μg dL^–1^ and presented in units of ppm in solution (Figure S4).

#### 2.3.3 Swelling Ratio

Optimal silicone oil uptake by the polymer matrix was evaluated through swelling ratio studies. To determine the optimal oil viscosity for subsequent studies, 10 and 50 centistoke (cSt) oil was selected based on optimization of previously reported materials.^29^ The initial mass of samples (M_0_) was recorded, and then they were incubated in silicone oil for 24 h. At various time points (1, 2, 3, 4, 5, 6, 24 h), samples were removed from the oil, and the mass was recorded (M_i_) for quantification of the swelling ratio profile. Final swelling ratio at each time point was calculated using eq 1.

$$\mathrm{Swelling Ratio}=\frac{M_{i}}{M_{0}}$$ 1

#### 2.3.4 Sliding Angle Measurements

Antifouling properties of the slippery surface modification introduced by silicone oil infusion were characterized by sliding angle testing on samples. For analysis, a 5 μL water droplet was introduced on the surface of a 12 mm sample disk attached to a glass slide. Following the introduction of the water droplet, the glass slide containing the sample was tilted on the stage of an Ossila contact angle goniometer (Sheffield, UK), and a 10 s, 200 frame video was captured and assessed via a frame-by-frame analysis in ImageJ to capture the sliding angle of the water droplet on the sample. Sliding angle stability was measured across 7 days with sample incubation in PBS at 37 °C at 150 rpm between each time point.

#### 2.3.5 Nitric Oxide Release

Real time NO release kinetics from the SNAP Cu LI samples were analyzed using a gold standard chemiluminescence method of quantification implementing a Zysense Nitric Oxide Analyzer (NOA 280i).^30^ During experimentation, 6 mm film disk samples were submerged in 3 mL of PBS at 37 °C in an amber vial to prevent external catalysis of SNAP from light. A nitrogen sweep gas facilitates the transport of released NO into the instrument, where a chemiluminescent reaction with photoexcited NO_2_ reacts with ozone (O_3_) releasing a photon and computing NO release in real-time at a ppb reading. This reading was normalized to NO release rate per unit surface area and reported as flux units (×10^–10^ mol min^–1^cm^–2^).

### 2.4 *In Vitro* Biological Testing of SNAP Cu LI

#### 2.4.1 24 h Bacterial Adhesion Assessment

The antibacterial efficacy of SNAP Cu LI was assessed using a 24-h bacterial adhesion assay. An overnight culture of *E. coli* (ATCC 25922) and MRSA (ATCC BAA41) in LB broth was grown at 37 °C at 150 rpm. The culture was centrifuged at 4400 rpm for 7 min to obtain a bacterial pellet, which was then resuspended in sterile 1× PBS. The bacterial suspension was diluted to 0.1 OD_600_ and added to a well plate containing 6 mm sample disks that were sterilized using UV sterilization for 15 min on each side. Samples were exposed to the bacterial solution for 24 h in 37 °C at 150 rpm to mimic dynamic physiological conditions. After exposure, samples were removed from bacterial solutions and gently rinsed with sterile 1× PBS to remove unadhered cells and homogenized at 25,000 rpm using an Omni THb homogenizer for 60 s, followed by vortexing for 60 s to detach adhered bacteria. Homogenization solutions were serially diluted and grown on LB agar plates overnight at 37 °C. After growth, the colony forming units were analyzed and normalized to the surface area of the sample (CFU cm^–2^).

#### 2.4.2 Cytocompatibility Assay

The biotolerability of SNAP Cu LI was studied following ISO 10993-5 standards for the biological evaluation of medical devices with extract testing against NIH/3T3 (ATCC CRL-1658) mouse fibroblast cells.^31^ 3T3 cells were revived from cryopreserved stocks and grown in DMEM media supplemented with 10% FBS and 1% penicillin–streptomycin (P/S) under a 5% CO_2_-humidified atmosphere at 37 °C. Cells were subcultured for up to 10 passages between experiments.

Studies were initiated by harvesting 3T3 cells at ∼70% subconfluency via enzymatic detachment with trypsin for 5 min, followed by centrifugation (200 rcf, 5 min), resuspension of the cell pellet, and quantification via trypan blue staining with a NanoEntek EVE cell counter. 3T3 cells were seeded into 96-well plates at a density of 5,000 cells well^–1^ on a basis of 100 μL well^–1^. Plates were grown for 24 h prior to treatment.

Extracts from SNAP Cu LI and related formulations were collected using the complete DMEM media with FBS and P/S as the extract vehicle. Circular coupons with the various modifications were incubated in a vehicle for 24 h at 37 °C at a standard surface-area-to-volume ratio of 1 cm^2^ mL^–1^. Afterward, culture media were aspirated from wells, and each collected extract was plated neat in triplicate (100 μL well^–1^). Untreated controls included wells supplemented with the clean vehicle. Cells were incubated for an additional 24 h; afterward, media was aspirated from wells and replaced with MTT (0.5 mg mL^–1^) in PBS (1×). Plates were incubated for an additional 3 h. Finally, supernatants were decanted, and remaining formazan salts dissolved in DMSO (100 μL well^–1^) and read for absorbance (λ = 570 nm) with background correction (λ_ref_ = 690 nm). Relative viability of treated groups was calculated according to eq 2. Final results are reported as mean cellular viability obtained from four independent extraction experiments per substrate type ± standard deviation.

$$\mathrm{Percent Cell Viability}=\frac{\Delta {\mathrm{ABS}}_{\mathrm{Sample}}}{\Delta {\mathrm{ABS}}_{\mathrm{Untreated}}}\times 100\%$$ 2

#### 2.4.3 Surface Repellence against Fibrinogen

The resistance of SNAP Cu LI substrates and related materials to surface fouling by plasma proteins was evaluated by measuring the surface adsorption of fluorescently tagged fibrinogen (FITC-Fg) following prior methods at a representative serum concentration of 2 mg mL^–1^ over 90 min at 37 °C.^32^ Substrates (6 mm, surface area of ∼0.28 cm^2^) were placed within 96-well plates and incubated with diluted FITC-labeled Fg 1:10 with unlabeled Fg (100 μL well^–1^). Following incubations, substrates were further washed with 100 μL equal volume of PBS (1×) to detach unbound Fg. Adsorbed FITC-Fg was quantified via fluorescence (Ex. 495; Em. 519) with the calculation of amounts of FITC-Fg (μg) with respect to a standard curve of known concentrations. Final results are reported as mean adsorbed Fg normalized to surface area (μg cm^–2^) ± standard deviation (*n* = 6).

#### 2.4.4 Platelet Adhesion Assessment

The fabricated surface was exposed to porcine blood platelet rich plasma (PRP) with a standardized concentration of platelets to assess surface platelet adhesion. Freshly drawn porcine blood was anticoagulated with sodium citrate at a ratio of 9:1. Anticoagulated whole blood was then centrifuged at 300 RCF for 12 min. The platelet-rich plasma (PRP) portion was pipetted out without disturbing the buffy coat layer of the leukocytes. The remaining blood was spun again at 3000 RCF for 20 min to collect platelet-poor plasma (PPP). The total platelet count of the PRP was determined using a HESKA Element-HT5 Hematology Analyzer (Loveland, CO, USA). The PRP was then diluted to 2 × 10^8^ platelets mL^–1^ with the PPP solution obtained earlier. Right before polymer incubation in the normalized platelet solution, calcium chloride (2.5 mM) was added to reverse the anticoagulant (sodium citrate), hence the working solution. An individual polymer (6 mm diameter) was exposed to 1 mL of the working platelet solution. Samples were then incubated at 37 °C for 90 min on a rocker. Samples were manually rocked every 30 min to ensure the surfaces were fully submerged.

Following the incubation period, the samples were removed and rinsed with PBS. The surface-adhered platelets were then lysed with a 2% v/v Triton X-100 solution for 30 min. Afterward, the degree of platelet adhesion was quantified by lactate dehydrogenase released during lysis using a Roche Cytotoxicity Detection Kit (λ = 492 nm with λ_ref_ = 620 nm). A calibration curve was constructed using known dilutions of the final platelet solution. The number of platelets adhered to the sample surface was interpolated from the calibration curve.

#### 2.4.5 Hemolysis Testing

The hemolytic response of the SNAP Cu LI samples and its controls was tested according to ISO 10993-4 standards using the direct contact method.^33^ Porcine whole blood (WB) was freshly acquired from the UGA Swine Unit and immediately citrated with a sodium citrate dihydrate solution to prevent premature coagulation. The hemoglobin concentration [Hgb] was measured in the neat WB and diluted to a working concentration of 10 mg mL^–1^ with calcium and magnesium-free PBS (CMF-PBS). The SNAP Cu LI samples and its controls were incubated in the working concentration of dilute whole blood (DWB) according to an extraction ratio detailed in ISO 10993-12 standards^34^ for 3 h at 37 °C with manual inversions every 30 min. A positive control and blank were prepared by diluting the DWB with deionized water and CMF-PBS, respectively, and were incubated and manually inverted at the same intervals. High-density polyethylene (HDPE) coupons were prepared and incubated in the DWB in accordance with the aforementioned extraction ratio as the negative control, following similar incubation and inversion times. After incubation, the samples were removed from the DWB, and the DWB was centrifuged to separate the lysed blood cells in the supernatant from the intact blood cells and components at the bottom of the tube. The supernatant was then reacted with an equal volume of Drabkin’s reagent for 15 min at room temperature, and the absorbance was read on a BioTek Plate Reader (Winooski, VT) at 540 nm.

A standard curve was prepared by dissolving porcine Hgb in CMF-PBS, serially diluting, and reacting with an equal volume of Drabkin’s reagent for 15 min at room temperature. The absorbance was quantified at 540 nm. The concentration of the Hgb from the DWB exposed to samples was then calculated, and the % hemolysis was determined according to eq 3, where [Hgb]_sample_ is the DWB supernatant concentration of the Hgb after sample incubation, [Hgb]_baseline_ is the DWB supernatant concentration of Hgb without lysing agents (i.e., the blank), and [Hgb]_total_ is the total amount of Hgb in the DWB with lysing agents (i.e., the positive control). Finally, the hemolytic index was calculated using eq 4. The final results are reported as a mean ± standard deviation (n = 5, *p* < 0.001).

$$\mathrm{\% Hemolysis}=\frac{{\left[\mathrm{Hgb}\right]}_{\mathrm{sample}}-{\left[\mathrm{Hgb}\right]}_{\mathrm{baseline}}}{{\left[\mathrm{Hgb}\right]}_{\mathrm{total}}}\times 100$$ 3

$$\mathrm{Hemolytic Index}\left(\mathrm{HI}\right)={\mathrm{\% Hemolysis}}_{\mathrm{sample}}-{\mathrm{\% Hemolysis}}_{\mathrm{NegControl}}$$ 4

### 2.5 Statistical Analysis

All reported data are reported as mean ± standard deviation (mean ± SD). Data analysis was conducted using GraphPad Prism software (Version 10.2.2). An ordinary one-way ANOVA with Tukey’s test was utilized for statistical analysis where *p* < 0.05 indicates statistical significance across sample types.

## 3 Results and Discussion

### 3.1 Fabrication and Characterization of SNAP Cu LI

#### 3.1.1 Film Fabrication and Implementation of Surface Modifications

Nitric oxide donors are widely used in various applications, ranging from medical to antimicrobial and antifouling surfaces.^35,36^ Given their sensitivity to surface interactions and biological conditions, characterizing these molecules is essential for designing biocompatible materials with targeted properties. Additionally, donor molecules show amplified effects when paired with complementary surface modifications.^16,37^ These surface modifications provide multifunctionality, enhancing or adding antimicrobial and antithrombotic properties to the fabricated material. Prior literature elaborates on antimicrobial NO releasing materials with metal nanoparticle incorporation; however, their antifouling properties are widely limited due to the lack of implementation of surface modification strategies that allow for repellence of blood proteins such as fibrinogen.^19,20,38^ Therefore, this work explores the optimization of antimicrobial NO releasing materials and implementing antifouling properties to these platforms through the combination of NO donor molecule SNAP into a polymer surface, copper nanoparticles (40–60 nm), and infusion with silicone oil.

SNAP was synthesized by modifying previously reported methods: the precursor molecule NAP was nitrosated with a mixture of concentrated hydrochloric acid, sulfuric acid, sodium nitrite, and methanol.^28^ This synthesis yielded a green powdered product that allows for homogeneous blending in a polymer solution. To ensure the purity of the synthesized SNAP product prior to polymer incorporation, NMR spectroscopy was used, in which the distinctive chiral center peaks were integrated to determine >97% purity of the synthesized NO donor (Figure S1). The material was fabricated using Dowsil 3140 RTV silicone rubber, a clinically relevant silicone rubber substrate, and dissolved in tetrahydrofuran (THF) at a concentration of 250 mg mL^–1^. This polymer solution was blended with a specific concentration of SNAP (10 wt %) and then cast into a polytetrafluoroethylene (PTFE) mold, where it was allowed to cure to form the experimental film. To introduce copper onto the surface, the material was dip-coated with a specific concentration of copper nanoparticles and RTV solution in THF (3 wt %), which has been reported to be effective against bacterial adhesion without eliciting cytotoxic effects.^19^ The RTV-copper solution acts both as an adhesive for the nanoparticles to laminate onto the surface, in addition to creating a topcoat on the polymer to control NO release and SNAP leaching from the bulk polymer. To restrict SNAP leaching and direct SNAP interactions with Cu, three barrier coats of RTV were applied to the base polymer followed by one CuNP topcoat. To swell the oil in the polymer, the samples were placed into a vial containing silicone oil at two different viscosities (10 and 50 cSt) followed by 24 h incubation. The final result of this fabrication yields the proposed experimental film, which possesses NO releasing properties through the blending of the donor molecule SNAP, catalytic and enhanced antimicrobial properties through the coating of CuNPs, and antifouling characteristics through the uptake of silicone oil by LI **(** Figure 1A**)**. To optimize the material, two silicone oil viscosities were initially evaluated using swelling ratio studies to determine the optimal amount of oil to load into the polymer matrix. The results of these studies determined that the optimal viscosity for proceeding to subsequent studies was 10 cSt, as the swelling ratio for SNAP Cu LI was 2.7 ± 0.2 (Figure 1B), whereas 50 cSt yielded only 1.6 ± 0.2 (Figure S2). These values provided insight into the stability and oil-replenishing properties of the experimental film; therefore, a higher swelling ratio helps determine the optimal oil viscosity for LI surface modification.

**Figure 1:**
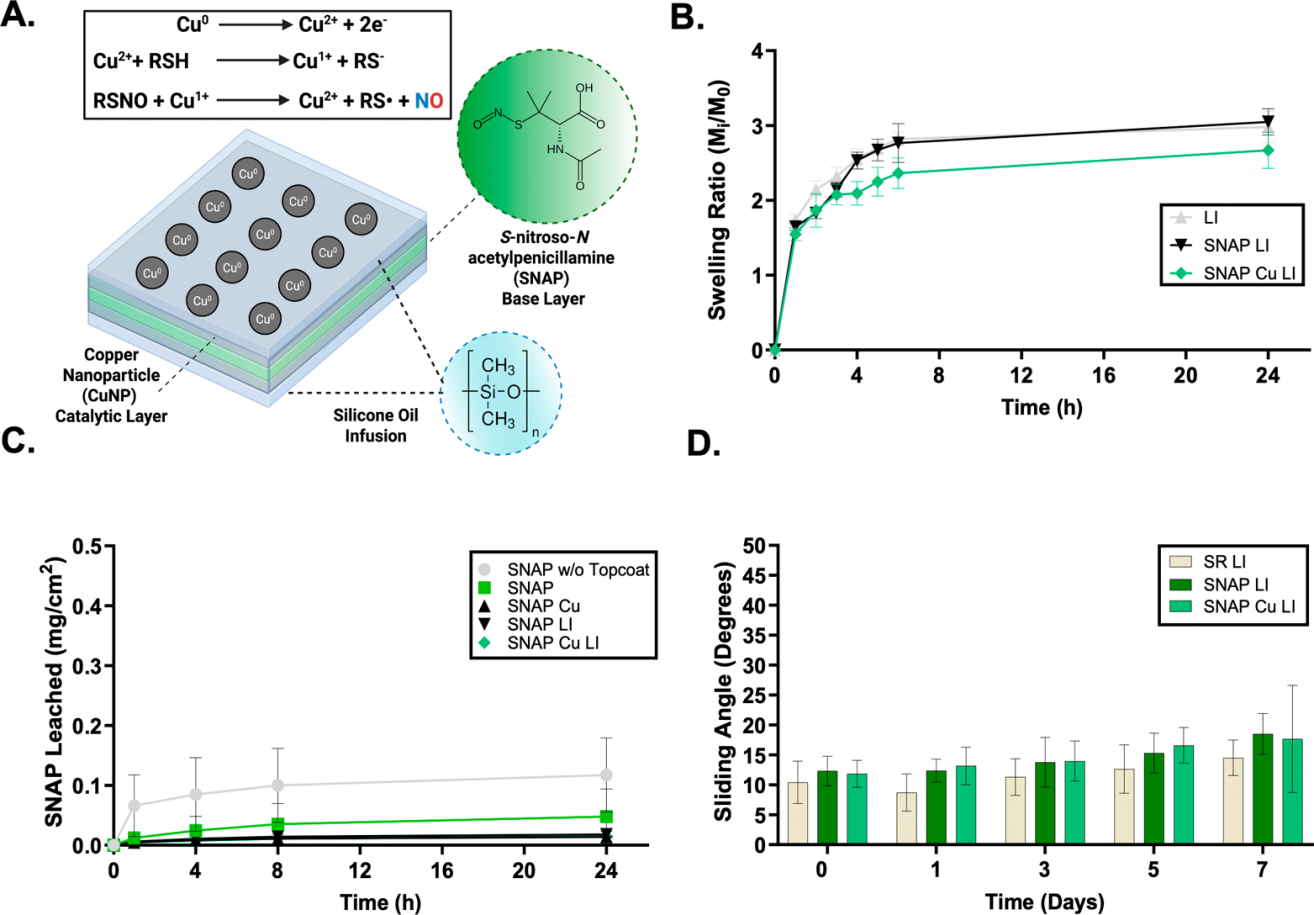
Proposed composition of SNAP Cu LI and nitric oxide generation mechanism of copper nanoparticles in the presence of RSNOs (A), swelling ratio studies of LI modified samples utilizing 10 cSt silicone oil (*n* = 4) (B), cumulative SNAP leaching of NO releasing samples across 24 h (*n* = 4) (C), and 7 day sliding angle stability study of LI containing samples (*n* = 10) (D). Data reported as mean ± SD.

#### 3.1.2 SNAP Leaching Analysis

Rapid leaching of the NO donor depletes the polymer’s NO reservoir too quickly, reducing the localized biological effects of nitric oxide. Although SNAP byproducts (i.e., NAP disulfides) and low concentrations of Cu are generally nontoxic, leaching studies are an essential step in film fabrication and crucial for understanding the transition into *in vitro* biological testing.^39,40^ In the proposed material, only NO should be selectively released through a catalytic cleavage of disulfide bonds between the donor molecule and stimuli such as heat, light, or Cu ions. Therefore, a 24-h SNAP leaching study was conducted to assess donor diffusion from the polymer matrix. SNAP leaching studies were performed by UV–vis spectroscopy in PBS for 24 h and compared to a standard curve of known SNAP concentrations (Figure S3). Samples were collected at 30 min, 1, 4, 8, and 24 h to quantify the cumulative leaching of the SNAP molecule (Figure 1C). This study was conducted by introducing a circular film of the fabricated material into phosphate-buffered saline (PBS) solution between each time point and the UV–vis reading of the solution at 340 nm. To prevent equilibrium saturation of the film and solution, the PBS was replaced at each time point, and a fresh solution was introduced after each reading. In this study, five sample types were assessed for SNAP leaching, as these combinations all incorporated SNAP into the polymer matrix. Based on the results of this study, the cumulative leaching profile of all NO-releasing films was less than 0.12 mg cm^–2^ over 24 h, consistent with biocompatible formulations of NO releasing substrates.^11,13,41^ These results are complemented by the combination of the proposed modifications as SNAP Cu LI exhibited negligible amounts of leaching (<0.01 mg cm^–2^) compared to other sample types including SNAP without topcoat and SNAP. This indicates the added topcoats and liquid infusion provide protective barrier coats from external moisture and water uptake that prevent donor leaching while still retaining ample permeability for NO release and desired biological properties.

#### 3.1.3 Sliding Angle Assessment

Blood proteins such as fibrinogen are among the earliest precursors of the blood coagulation cascade.^42,43^ The prevention of these proteins from adsorbing to the surfaces of polymeric materials is crucial for triggering and mitigating the coagulation cascade response, which involves platelet activation and thrombus formation. The stability of fabricated surface modifications is critical in preventing adsorption. Silicone oil is a stable lubricant for integration into polymeric interfaces due to its high chemical compatibility with polymers such as PDMS and its low surface energy, which prevents biofouling on the polymer surface.^26,29,44^ The proposed material was optimized using different viscosity oils (10 and 50 cSt), and it was determined that 10 cSt oil was loaded more efficiently. The stability of this LI surface modification under physiological conditions was assessed over 7 days. A sliding angle study assesses the slippery properties of a sample by tilting it. This was conducted to assess the material’s potential for antifouling properties following the LI layer modification. The material was evaluated using a contact angle goniometer by dispensing a 5 μL water droplet onto the surface and tilting it until the droplet slid off the polymer surface. The tilting/sliding angle was quantified frame by frame using ImageJ. The results indicate sustained surface slipperiness, with sliding angles below 20° over a 7-day period. SR LI maintained a sliding angle of 14.5 ± 3.0°, SNAP LI exhibited a sliding angle of 18.5 ± 3.4°, and SNAP Cu LI exhibited a sliding angle of 17.7 ± 9.0° after 7 days of submersion **(** Figure 1D**)**. Remarkably, despite the incorporation of surface-bound metal and morphological changes, the lubricity and slippery characteristics of SNAP Cu LI remained stable throughout extended dynamic testing, while nonslippery materials are reported to possess water pinning behaviors with sliding angles of >90°.^45^

#### 3.1.4 Nitric Oxide Release Kinetics

Nitric oxide (NO) is an endogenous gaseous free radical, and understanding its controlled release from a polymeric surface at physiologically relevant levels is essential for evaluating the material’s antimicrobial and antithrombotic performance.^46^ Previous reports explored nitric oxide release kinetics of materials combined with metal nanoparticles and liquid infusion; however, the evaluation of the combination of all these components remains to be explored.^13,19,20,29,47^ In this work, the SNAP Cu LI material was evaluated for its release profile using a chemiluminescence based method with a Sievers 280i Nitric Oxide Analyzer (NOA) over a 7 day period, demonstrating its stability to sustain NO release from the polymeric matrix.

This experiment involved placing the sample in a PBS solution and submerging it in a water bath at 37 °C to mimic physiological conditions. The NO release was normalized to the rate of NO flux to the surface area of the polymer film. The average physiological flux from endothelial cells is 0.5–4.0 × 10^–10^ mol min^–1^ cm^–2^; therefore, these levels provide a parameter for medical device applications that mimic physiological functions to improve biocompatibility.^7^ The catalytic NO release mechanism of this material is promoted through redox-mediated reactions of Cu^0^ nanoparticles undergoing oxidation to Cu^2+^ and reduction to Cu^1+^ in the presence of reactive species and S-nitrosothiols like SNAP (Figure 1A). Copper ions further catalyze the decomposition of SNAP to release NO and, when introduced as a surface modification, can enhance the substrate’s biological efficacy by improving antimicrobial activity and hemocompatibility **(** Figure 2A**)**. Although the metal itself possesses inherent antimicrobial properties, the elevated NO release from the film—catalyzed by interactions with the metal—further enhances these effects. In addition to its antimicrobial activity, the film exhibits enhanced antiplatelet activity. In this study, it is evident that these interactions are complementary, as at d0 CuNP coated samples exhibited a statistically significant elevation in NO release, with SNAP Cu having a flux of 2.4 ± 0.3 × 10^–10^ mol min^–1^ cm^–2^ and SNAP Cu LI exhibiting an NO flux of 1.8 ± 0.3 × 10^–10^ mol min^–1^ cm^–2^. Prior reports indicate that integration of LI modifications on polymeric materials provides a lower, more sustained release rate of NO from the substrate due to reduced water uptake of the material, during the first day of testing.^13,44^

**Figure 2:**
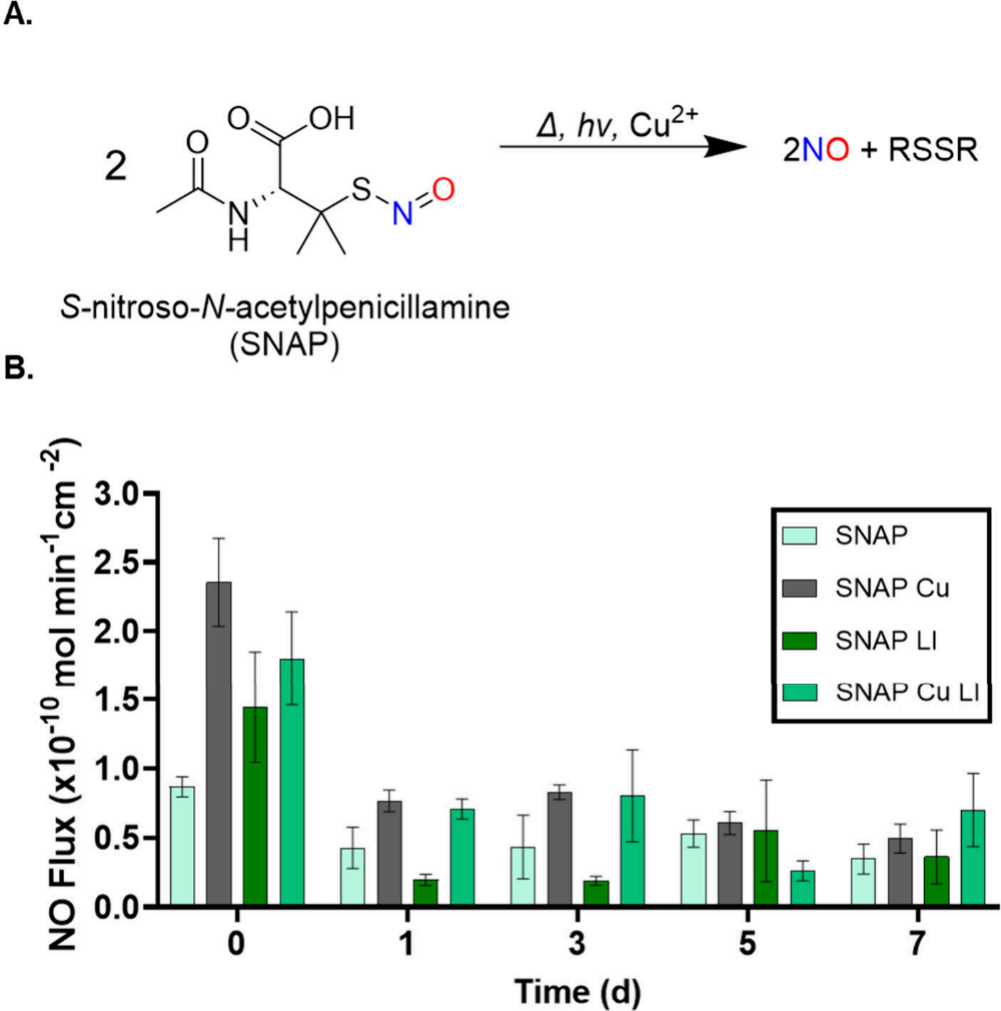
Decomposition of SNAP to release NO under physiological conditions and in the presence of heat, light, and metal catalysts (A). NO release kinetics of SNAP blended samples (B). Data reported as mean ± SD (*n* ≥ 3).

Notably, the interactions of the introduced LI layer do not significantly inhibit the release kinetics of NO through the polymer matrix or silicone oil modification. NO is a small gas that is highly permeable across hydrophobic interfaces, such as those between silicone oil and polymer matrices. Therefore, with the introduction of the LI surface modification, diffusion of NO is facilitated by the liquid-like interface that is present from the LI compared to a solid diffusion barrier.

This work aims to improve the tunability of the LI modification by mediating and enhancing NO release from the proposed material with the addition of CuNPs. This tunability and enhanced NO release further improve the antimicrobial properties of NO releasing materials while adding slippery antifouling behavior to these platforms. Compared to unmodified SNAP LI and SNAP, SNAP Cu LI exhibited sustained release of NO across the duration of the entire study where even at d7, the samples were still releasing NO at physiologically relevant levels with a flux of 0.7 ± 0.3 × 10^–10^ mol min^–1^ cm^–2^ which further supports the material’s property to efficacy at releasing NO with minimal donor leaching from the polymer matrix (Figure 2B).

The integration of the liquid infusion surface modification showed no significant inhibitory effect on NO release on the first day of testing, a crucial time point, as many HAIs are associated with bacterial infections during device implantation and with surgical site infections within the first few hours of contact.^48^ These studies will provide key insights into the rate of NO release and further the understanding of the materials’ properties.

### 3.2 Biological Evaluation

#### 3.2.1 *In Vitro* Antibacterial Efficacy Assessment

Surface fouling and infection caused by bacterial attachment to medical devices are among the leading causes of device failure and extended hospitalization in clinical settings.^49^ Specifically, strains such as methicillin-resistant *Staphylococcus aureus* and *Escherichia coli* burden medical surfaces and can undergo mechanisms that irreversibly attach these cells to surfaces, form extracellular polymeric substances (EPS), and ultimately lead to biofilm formation.^50^ Traditional therapies, such as antibiotic administration, can cause additional complications, including antimicrobial resistance (AMR), increased patient risk, and greater treatment difficulty.

Gaseous NO is a promising alternative for combating bacterial infections due to its nonspecific mechanism of action, its ability to eradicate bacterial cells, and its antibiofilm properties.^51^ Specifically, NO acts through various processes that affect cell membrane function and induce oxidative and nitrosative stress via reactions with molecular oxygen, producing reactive oxygen species (ROS) and reactive nitrogen species (RNS), such as peroxynitrite and dinitrogen tetroxide.^52^ These reactions induce cellular damage and death by cleaving genetic material, peroxidizing membrane lipids, and nitrosating membrane proteins. Copper nanoparticles also possess antibacterial properties that, when combined with NO-releasing platforms, enhance their efficacy against bacterial species. Alone, CuNPs induce ROS generation via Fenton-like reactions in bacterial cells, thereby causing cell damage and death.^53^ When used in combination with NO, elevated NO release from catalysis of donor degradation elevates ROS levels, respectively, which ultimately leads to more effective antibacterial properties. Specifically, these ROS and RNS, such as ONOO^–^ and N_2_O_3,_ can nitrosate reactive thiols and amines in membrane proteins, compromising their integrity and ultimately eradicating bacterial cells. In addition to these combinations, the addition of a passive surface modification, such as liquid infusion, reduces bacterial adhesion to surfaces.^54^ These NO-releasing, Cu-coated, slippery materials work by reducing capillary forces between the substrate surface and bacterial cells, thereby preventing surface fouling and infection.^55^

In this study, nitric oxide releasing polymers with CuNP and LI topcoats were formulated to simultaneously combat bacterial infections and prevent surface fouling caused by these microorganisms. To evaluate bacterial-killing efficacy, a 24-h bacterial adhesion assay was performed. Implantation infection is a precursor to device failure and patient complications; therefore, assessing this material’s immediate effects at a 24-h time point is crucial for addressing clinical infection rates.^56^ For these studies, two clinically relevant Gram-negative and Gram-positive strains were selected for testing the material. When exposed to the antibiotic-resistant species MRSA, the SNAP Cu LI surface exhibited a 2.7 log reduction (99.8%) in viable CFU cm^–2^ when compared to SR control and a statistically significant difference against other NO-releasing combinations (Figure 3A, Table S2). SNAP Cu LI was also exceptionally effective against Gram-negative species, achieving a 1.9 log reduction (98.7%) in viable *E. coli* cells (Figure 3B, Table S3). This combination of surface modifications also showed a statistically significant reduction in bacterial cells compared with other sample combinations, such as SNAP, SNAP Cu, and SNAP LI, further demonstrating the antifouling and antibacterial effects of the surface modifications. These results indicate that SNAP Cu LI surfaces have the potential to address clinical complications associated with bacterial infection, based on their mechanism of eradication and adhesion prevention.

**Figure 3:**
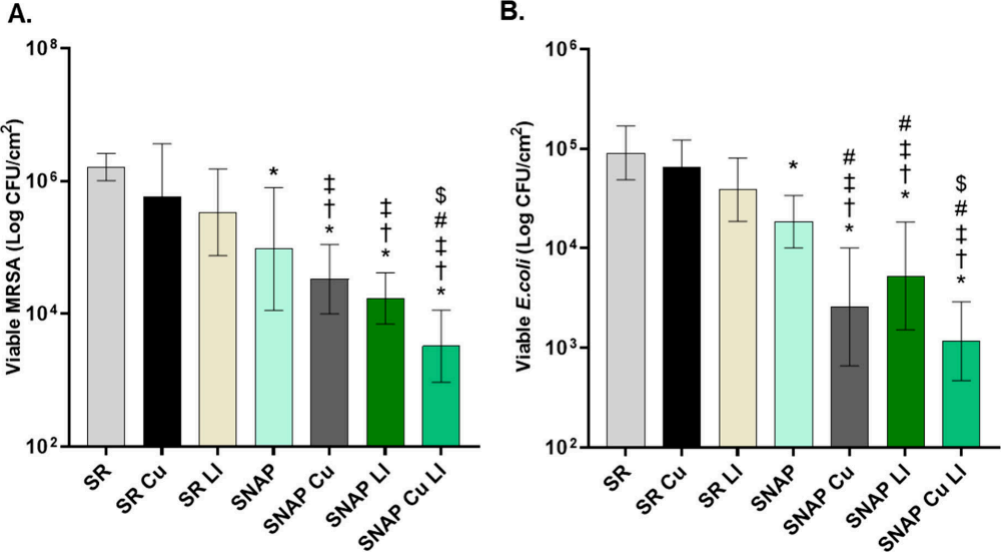
24-h bacterial adhesion assessment on SNAP Cu LI against *E. coli* ATCC 25922 (A) and MRSA BAA41 (B) where * indicates *p* < 0.05 vs SR, † indicates *p* < 0.05 vs SR Cu, ‡ indicates *p* < 0.05 vs SR LI, # indicates *p* < 0.05 vs SNAP, and $ indicates *p* < 0.05 vs SNAP LI. Data are reported as mean ± SD (*n* ≥ 8).

#### 3.2.2 *In Vitro* Cytocompatibility Analysis

Biocompatibility evaluation was performed in accordance with ISO 10993-5, with extracts collected over 24 h at 37 °C in serum-supplemented medium. In general, ISO standardized criteria were met for extracts from all substrate classifications (defined as >70% relative viability retained relative to the blank vehicle), although SR Cu exhibited a trend toward toxicity (Figure 4). This result is consistent with an upward trend in Cu ion leaching (Figure S5, Table S1). Notably, these effects are balanced by LI surface modifications, which regulate diffusion and nonspecific leaching of Cu^2+^ ions into the physiological environment, and by the low levels of NO release in SNAP samples, which support fibroblast proliferation.^57^ Cu^2+^ is a potent agent that can impart toxicity to fibroblasts at concentrations on the order of 200 μM, whereas it confers orders-of-magnitude higher reduction rates against bacteria.^58^ Therefore, it is critical for surface designs with copper catalytic components to control the degradation/diffusion of CuNPs and their ion products to reduce off-target toxicity. The liquid lubricant silicone oil is similar to other materials used in clinically available implants and exhibits minimal toxicity toward 3T3 cells (Figure 4).^59^ The final SNAP Cu LI construct exhibited an upward trend in viability (102.9% ± 9.4%, see Figure 4), supporting the general tolerability of the surface design strategy for further biological evaluation.

**Figure 4:**
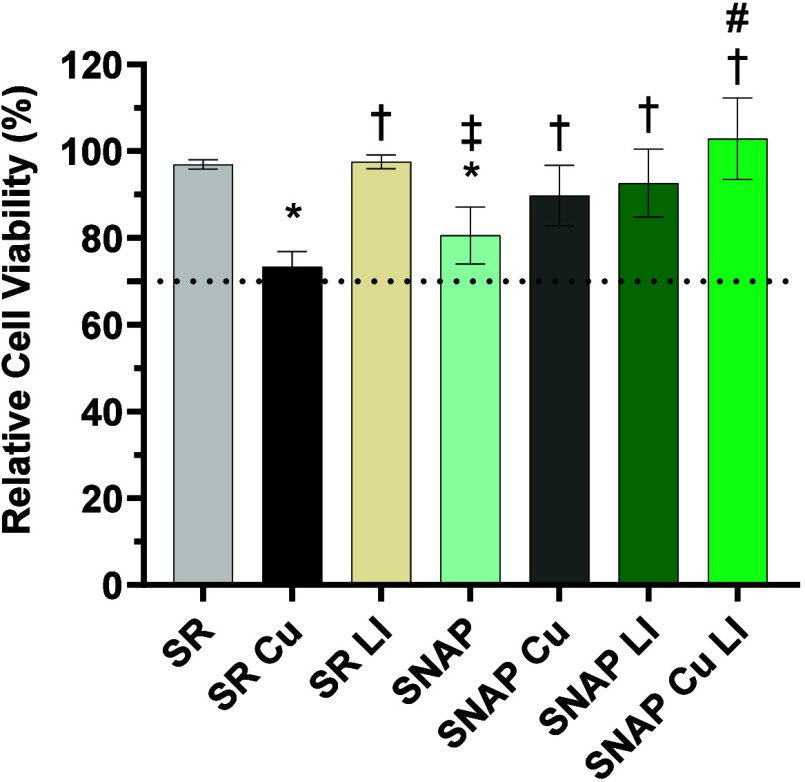
Cytocompatibility evaluation of SNAP Cu LI in a 24-h leachate MTT assay using NIH 3T3 Fibroblast cells. * indicates *p* < 0.05 vs SR, † indicates *p* < 0.05 vs SR Cu, ‡ indicates *p* < 0.05 vs SR LI, # indicates *p* < 0.05 vs SNAP. Data are reported as mean ± SD (*n* = 4).

#### 3.2.3 Antifouling Performance against Blood Proteins

A key component of developing blood-contacting biomaterials is optimizing minimally invasive surface modification strategies that repel biofoulants. One of the precursor triggers of the blood coagulation cascade involves the recruitment of blood proteins, such as fibrinogen, which convert to fibrin via enzymatic cleavage by thrombin. In turn, this promotes platelet activation and the formation of a thrombus.^60^ Although NO release from polymeric materials attenuates the activation of platelets on the surface, increased fibrinogen adsorption remains a limitation of these strategies.^42,61^ Therefore, developing a surface that prevents the adsorption of these proteins opens new avenues for the development of hemocompatible medical devices.

In this study, the antifouling properties of the SNAP Cu LI surface strategy and related formulations were evaluated by incubating them with the plasma protein fibrinogen as a metric of resistance to biofouling. Hydrophobic surfaces, such as SR, facilitate rapid protein deposition in blood-contacting environments, creating anchoring sites for platelet adhesion/activation and, ultimately, thrombus formation (Figure 5A).^42^ Liquid infusion facilitates a lubricant interface that has been shown to mediate physiological fluid interaction, resulting in little to no plasma protein adsorption (Figure 5B).^59^

**Figure 5:**
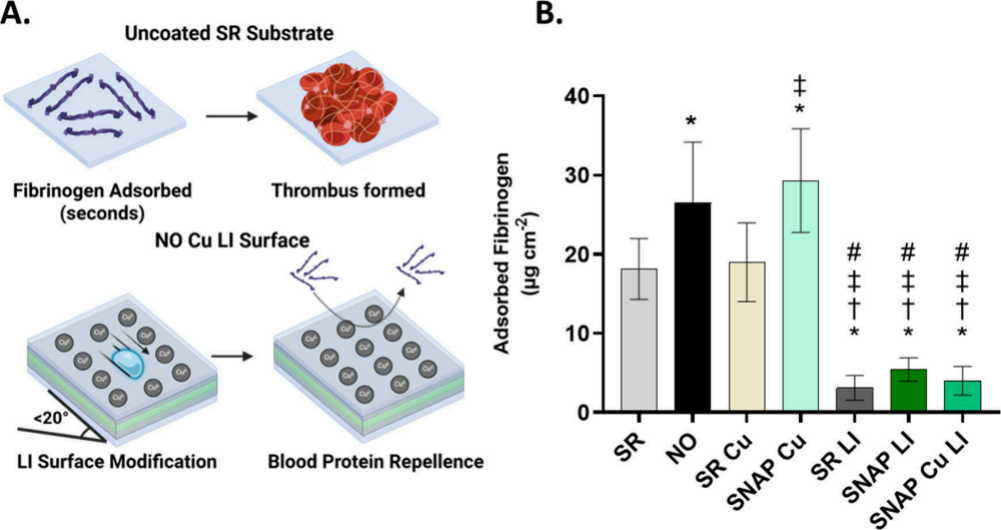
Fouling mechanism of blood proteins in seconds on uncoated device substrates versus antifouling properties of SNAP Cu LI against fibrinogen (A). Quantified protein adsorption studies on LI surface modification normalized to the surface area of samples (μg cm^–2^) (B). * indicates *p* < 0.05 vs SR, † indicates *p* < 0.05 vs SNAP, ‡ indicates *p* < 0.05 vs SR Cu, # indicates *p* < 0.05 vs SNAP Cu. Data are reported as mean ± SD (*n* ≥ 6).

In prior literature reports and herein, NO induces protein nitrosylation, leading to conformational changes. This is known to result in increased Fg deposition on SNAP surfaces compared to SR controls (Figure 5B).^62^ Plasma protein interactions with NO are known to increase aggregate deposition on NO-releasing surfaces, necessitating a complementary passivation strategy to achieve robust antibiofouling properties.^62^ Incorporation of the liquid infusion strategy into silicone rubber (SR LI) led to a ∼82.8% reduction in adsorbed Fg compared with SR controls, with comparable reductions achieved with both SNAP LI and SNAP Cu LI formulations (Figure 5B). These findings support the efficacy of the lubricant interface in complementing the NO-releasing and catalytic components of the embedded SNAP and Cu NP composite layers to facilitate desired antibiofouling effects.

#### 3.2.4 Prevention of Platelet Activation

After blood proteins adhere to a foreign surface, they undergo conformational changes that activate factor XII of the intrinsic pathway of the coagulation cascade. The activation of this factor recruits platelets, which then activate and aggregate on the material surface.^63^ The aggregation further leads to the formation of thrombi, which can detach and lead to further clinical complications caused by device failure, such as embolism and stroke.^64^ Developing a surface that minimizes the triggering of this cascade and even the initial attachment of proteins can further pave a path toward improving hemocompatible materials (Figure 6A). As shown in protein adsorption studies, LI surface modification can significantly reduce protein adsorption onto the device surface. Blood protein fibrinogen is a key component of platelet-driven coagulation as it binds to GPIIb/IIIa complexes to bridge platelets and promote aggregation.^65^ Furthermore, NO released from SNAP also acts as an agent that prevents surface-induced platelet activation.^66^ The multifunctional design of SNAP Cu LI materials is a mechanistically viable strategy for suppressing platelet activation pathways and thrombus formation at various stages of the coagulation cascade.

**Figure 6:**
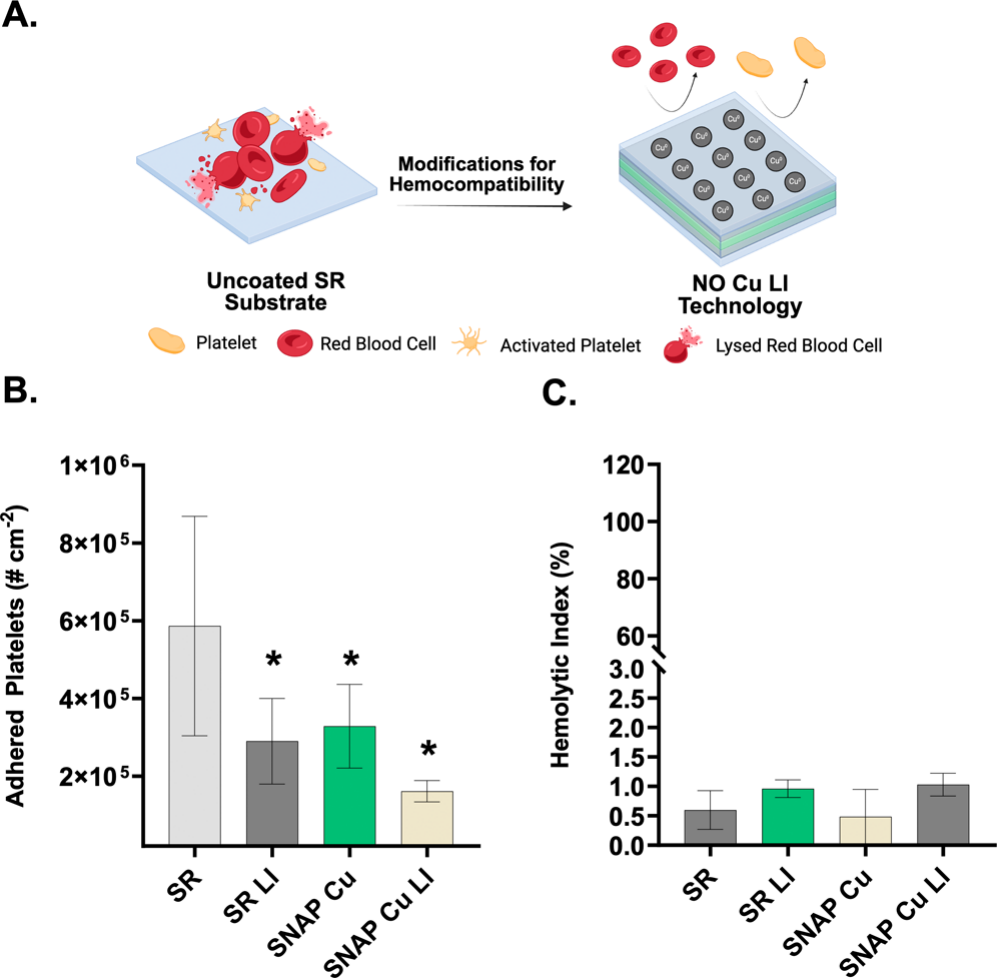
Platelet activation and hemolytic activity on uncoated SR substrate versus antiplatelet and hemocompatible properties of SNAP Cu LI (A). Percent reduction in platelet activation compared to SR (B) (*n* ≥ 4). Hemolytic activity of samples exposed to whole porcine blood (C) (*n* = 5). * indicates *p* < 0.05 vs SR. Data are reported as mean ± SD.

In this work, a lactate dehydrogenase (LDH) assay was employed, where samples were exposed to blood platelets, and platelet activation was quantified by measuring the adherence of activated platelets to the surface.^67^ In tandem with these tests, blood cell lysis was also conducted to ensure that the material is hemocompatible. SR, SR LI, SNAP Cu, and SNAP Cu LI samples were incubated in fresh porcine platelet-rich plasma for 90 min at 37 °C with gentle rocking. Subsequently, the adhered platelets were detached from the surface using lysing buffer and quantified. Both SR LI and SNAP Cu reduced the amount of adhered platelets by 50.6% and 44.0%, respectively. SNAP Cu LI samples significantly reduced platelet adhesion by 72.5% compared to the control (Figure 6B). This data show that SNAP Cu LI has the potential to not only inhibit adsorption of a key blood protein responsible for thrombus formation but also attenuate platelet activation and adhesion, consistent with other NO-releasing hemocompatible materials.^20,68,69^ In addition to platelet activation assays, hemolysis assays quantify the percentage of blood cells lysed upon exposure to the test films. The results from this study indicate less than 2% hemolysis across all tested sample types (Figure 6C). These results indicate that the material is nonhemolytic according to ISO 10993-5 protocols.^70^ The results of these studies indicate that the material is hemocompatible, which provides key insight into how these materials can be translated to clinical applications.

## 4 Conclusions

The fabricated material aims to address preeminent challenges in the clinical setting related to device complications. The novel combination of the NO donor molecule SNAP with CuNPs and LI creates a surface with broad-spectrum multifunctionality that addresses multiple complications arising from device implantation and medical procedures. SNAP and CuNP combinations address bacterial infection and platelet activation, whereas liquid infusion imparts a slippery, ultralow-fouling surface that further enhances antibacterial activity and prevents initial adsorption of blood proteins onto the device surface. These results and proposed technology can be applied to a broad-spectrum of indwelling medical device applications. These include blood-contacting and antibacterial materials such as central venous catheters (CVCs), extracorporeal circuits (ECC), and urinary catheter platforms.

SNAP Cu LI materials displayed effective stability properties and biocompatibility *in vitro.* In a NO release kinetics study, the combination of SNAP Cu LI released physiologically relevant levels of NO over 7 days, with a flux of 0.7 ± 0.3 × 10^–10^ mol min^–1^ cm^–2^. These materials were also effective against MRSA and *E. coli*, reducing them by over 99% and 98.7%, respectively. Fibrinogen adsorption studies demonstrated the antifouling nature of the slippery surface modification, with >80% reduction in protein adsorption on SNAP Cu LI compared with a SR control. Hemocompatibility properties were assessed, and more than 72% reduction in platelet activation and less than 2% hemolysis on the polymer surface were observed. In conclusion, the novel combination of multiple surface modifications, including NO catalysis with CuNPs and LI, endows the material with the potential to address issues related to bacterial infection, surface fouling by blood proteins, and thrombosis.

## Supplementary Material



## Notes

SNAP purity using ^1^H NMR spectroscopy; 24 h swelling ratio profiles of LI samples using 50 cSt silicone oil; atandard curve of known concentrations of SNAP in phosphate-buffered saline (PBS); standard curve of Cu^2+^ leaching in PBS using copper assay kit; Cu^2+^ leaching in PBS using copper assay kit; calculated p-values of the 24 h MRSA bacterial adhesion study comparing each sample type to each other; calculated p-values of the 24 h *E. coli* bacterial adhesion study comparing each sample type to each other (PDF)
