# Supplementary material for: Combinatorial Design of Slippery, Nitric Oxide-Releasing Surfaces Incorporating Copper Nanoparticles for Blood-Contacting Devices
Source: ACS Appl Mater Interfaces. 2026 Apr 27;18(17):24147–58. doi: 10.1021/acsami.6c02385 (PMC13154129; doi:10.1021/acsami.6c02385)
Supplement: Supplementary file 1 [file am6c02385_si_001.pdf]

SUPPORTING INFORMATION

Combinatorial Design of Slippery, Nitric Oxide–Releasing Surfaces Incorporating Copper Nanoparticles for Blood-Contacting Devices

Vicente D. Pinon[‡], Mark R.S. Garren[†], Yi Wu[†], Grace H. Nguyen[†], Elizabeth J. Brisbois[†], Hitesh Handa[‡†*]

[‡]Department of Pharmaceutical & Biomedical Sciences, University of Georgia

[†]School of Chemical, Materials, and Biomedical Engineering, College of Engineering, University of Georgia

[†*]Corresponding Author

Dr. Hitesh Handa

Professor

University of Georgia

I-STEM-2 Rm 2230

302 East Campus Rd,

Athens, GA 30606

Telephone: (706) 542-8839

E-mail: hhanda@uga.edu

Supporting Figures

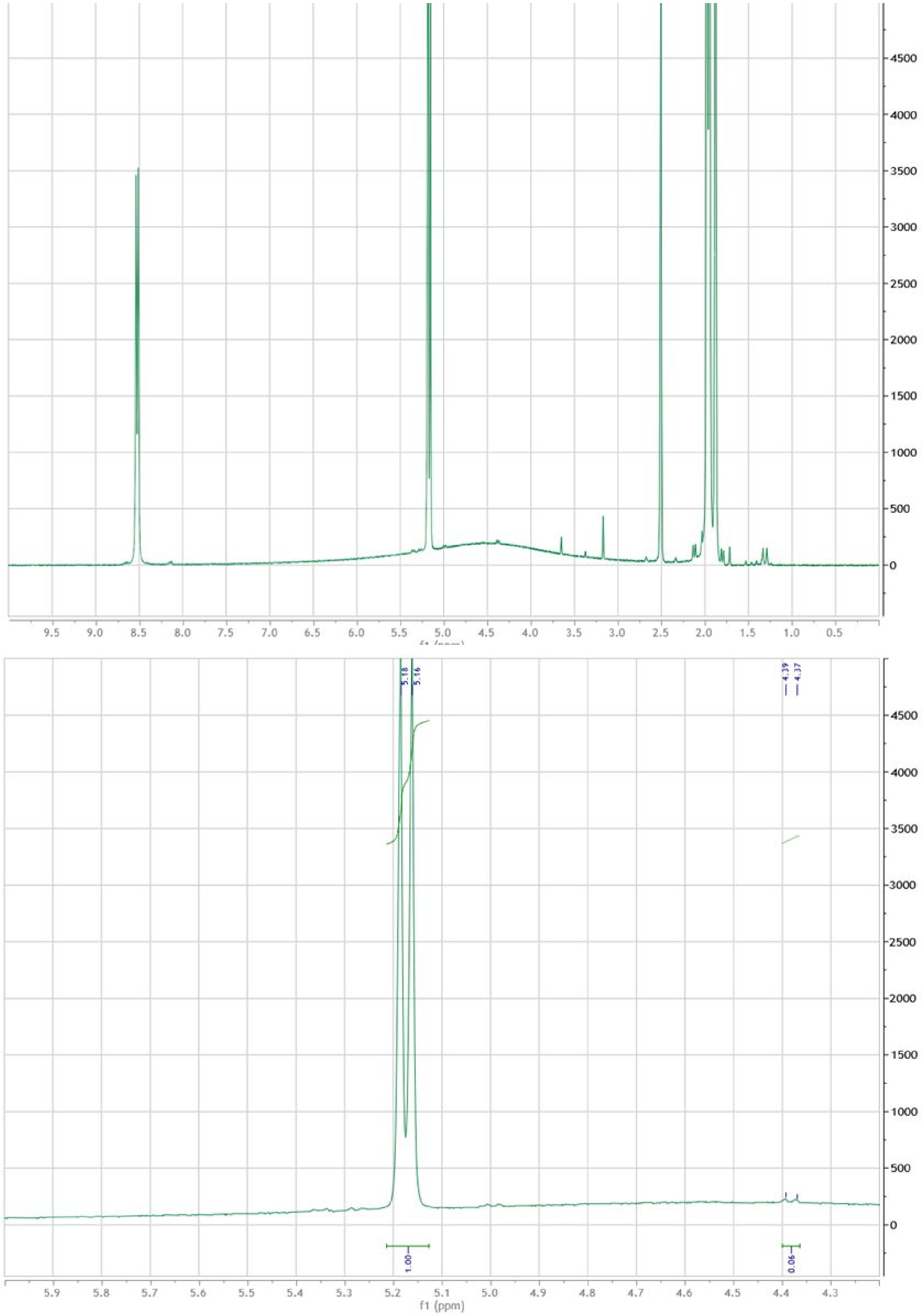

Figure S1: SNAP Purity using ^{1}H NMR spectroscopy.

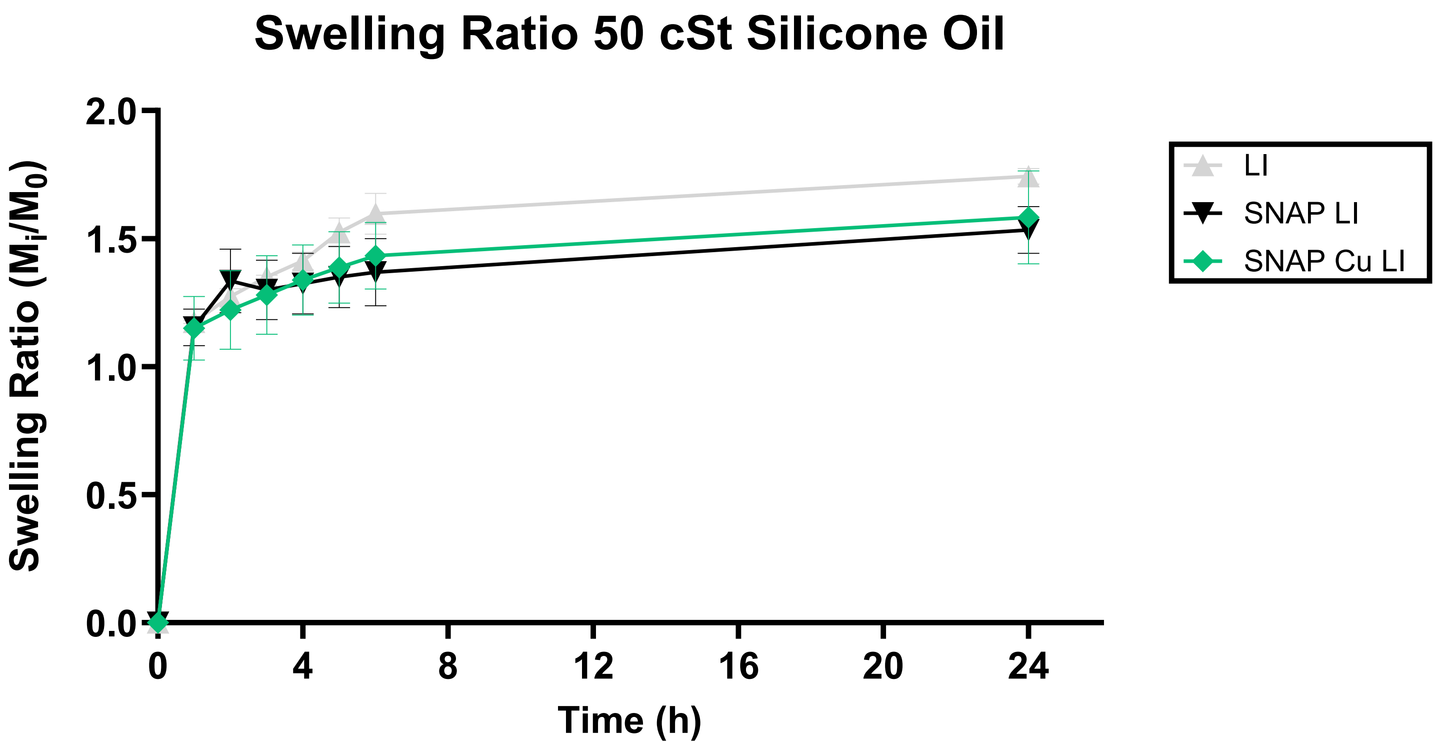

Figure S2: 24 hour swelling ratio profiles of LI samples using 50 cSt silicone oil. Data represented as mean +/- SD (n=4).

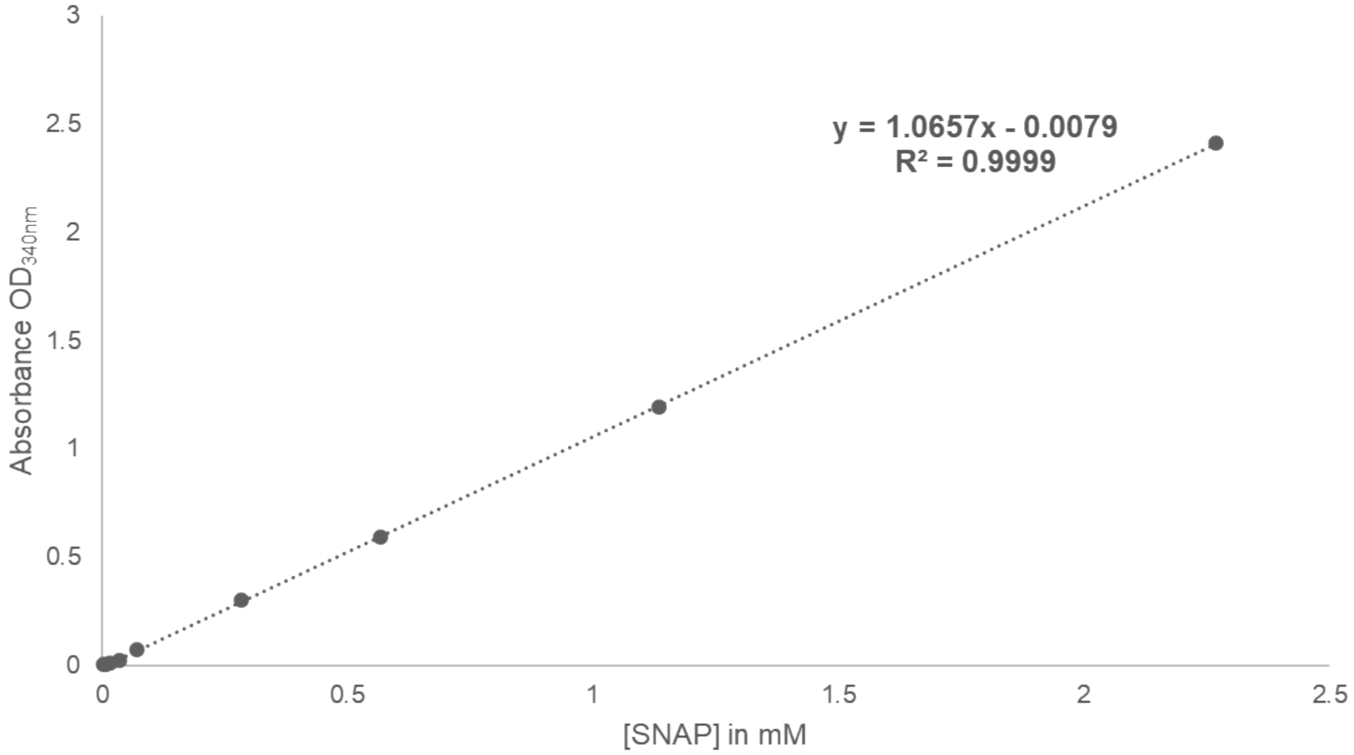

Figure S3: Standard curve of known concentrations of SNAP in phosphate buffered saline (PBS). Absorbance taken at wavelength of SNAP (340nm).

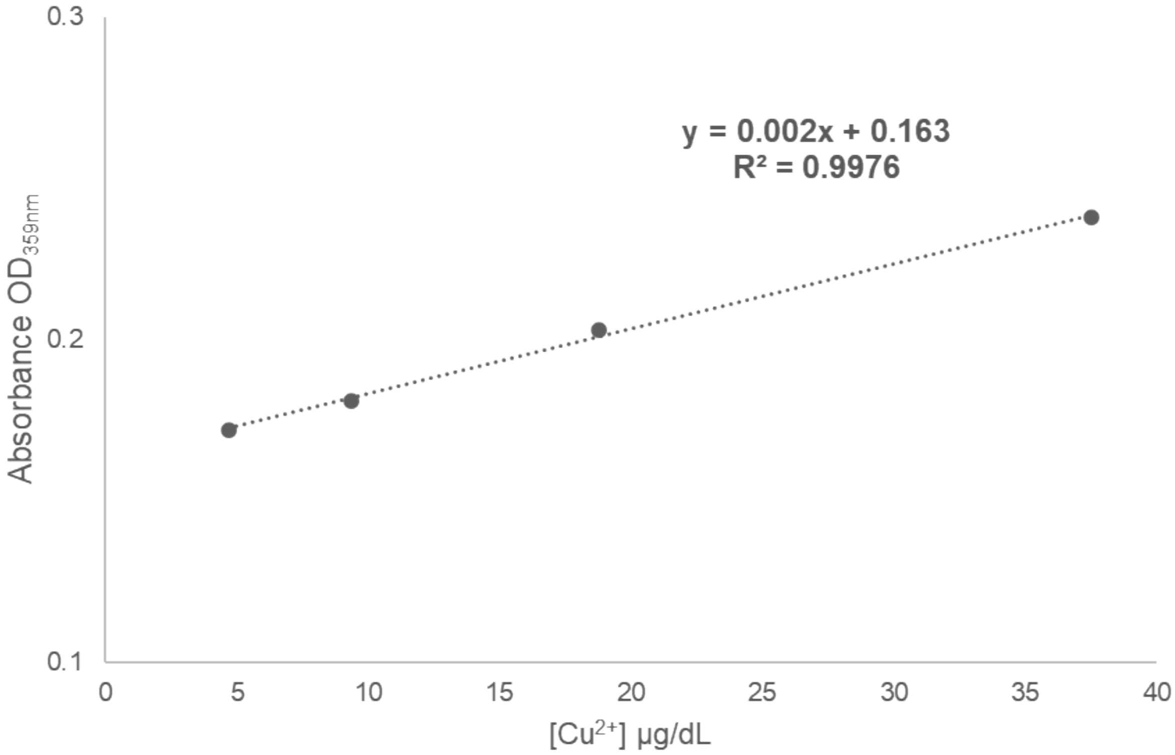

Figure S4: Standard curve of Cu^{2+} leaching in PBS using Copper Assay Kit (Sigma Aldrich, St. Louis, MO). Absorbance readings were taken at 359nm.

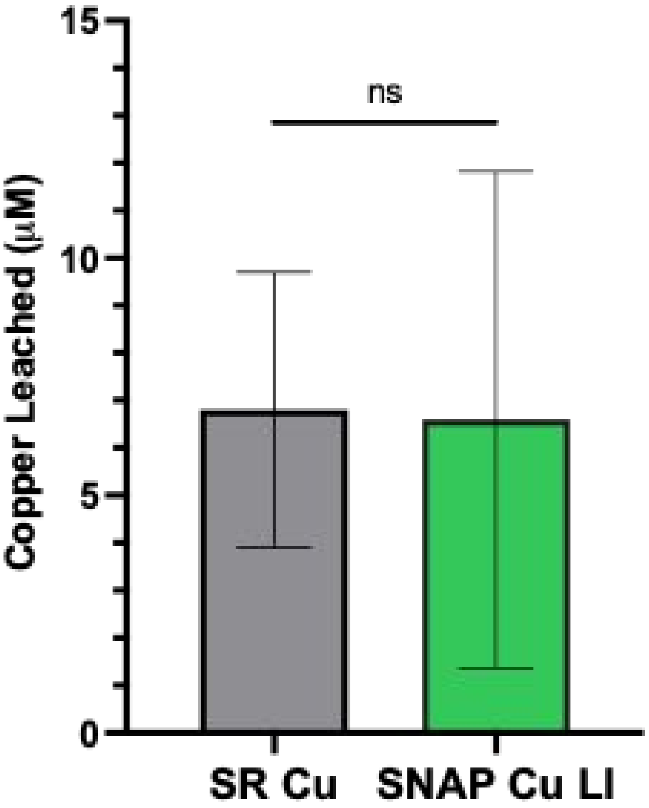

Figure S5: Cu^{2+} leaching in PBS using Copper Assay Kit (Sigma Aldrich, St. Louis, MO). Absorbance readings were taken at 359nm.

Supporting Tables

Table S1: Copper leaching from experimental samples using the Copper Kit (Sigma Aldrich, St. Louis, MO) colorimetric assay.

SR Cu	
Concentration in PPM	μM
0.22	3.51
0.505	7.95
0.569	8.96

SNAP Cu LI	
Concentration in PPM	μM
0.248	3.90
0.208	3.27
0.802	12.62

Table S2: Calculated p-values of 24 h MRSA bacterial adhesion study comparing each sample type to each other.

Sample Type	SR	SR Cu	SR LI	SNAP	SNAP Cu	SNAP LI	SNAP Cu LI
SR	-	0.7684	0.3135	0.0039	<0.0001	<0.0001	<0.0001
SR Cu	0.7684	-	0.9889	0.1712	0.0038	0.0002	<0.0001
SR LI	0.3135	0.9889	-	0.5641	0.0316	0.0021	<0.0001
SNAP	0.0039	0.1712	0.5641	-	0.7613	0.2201	0.0004
SNAP Cu	<0.0001	0.0038	0.0316	0.7613	-	0.9654	0.0309
SNAP LI	<0.0001	0.0002	0.0021	0.2201	0.9654	-	0.2510
SNAP Cu LI	<0.0001	<0.0001	<0.0001	0.0004	0.0309	0.2510	-

Table S3: Calculated p-values of 24 h *E.coli* bacterial adhesion study comparing each sample type to each other.

Sample Type	SR	SR Cu	SR LI	SNAP	SNAP Cu	SNAP LI	SNAP Cu LI
**SR**	-	**0.9535**	**0.2236**	**0.0020**	**<0.0001**	**<0.0001**	**<0.0001**
**SR Cu**	**0.9535**	-	**0.7733**	**0.0291**	**<0.0001**	**<0.0001**	**<0.0001**
**SR LI**	**0.2236**	**0.7733**	-	**0.5606**	**<0.0001**	**<0.0001**	**<0.0001**
**SNAP**	**0.0020**	**0.0291**	**0.5606**	-	**<0.0001**	**0.0347**	**<0.0001**
**SNAP Cu**	**<0.0001**	**<0.0001**	**<0.0001**	**<0.0001**	-	**0.3672**	**0.0309**
**SNAP LI**	**<0.0001**	**<0.0001**	**<0.0001**	**0.0347**	**0.3672**	-	**0.0074**
**SNAP Cu LI**	**<0.0001**	**<0.0001**	**<0.0001**	**<0.0001**	**0.0309**	**0.0074**	-
